# Supplementary material for: Analysis of PPARγ Signaling Activity in Psoriasis
Source: Int J Mol Sci. 2021 Aug 10;22(16):8603. doi: 10.3390/ijms22168603 (PMC8395241; doi:10.3390/ijms22168603)
Supplement: Supplementary file 1 [file ijms-22-08603-s001.zip › Supplemental materials_Analysis of PPARg signaling activity in psoriasis/Pathway models/Models images and html files/Model of PPARG related pathways in psoriasis (short version)/258234.html]

FICZ


# Small Molecule FICZ

|  |  |
| --- | --- |
| URN | urn:agi-cas:172922-91-7 |
| Total Entities | 0 |
| Connectivity | 332 |
| Name | FICZ |
| Molecular Weight | 282.301000 |
| XLogP | 3.990000 |

---

|  |  |
| --- | --- |
| Pathway | AHR Signaling in Th17 Cells Function |
|  | AHR Signaling in Tr1 Cells Function |
|  | Aryl Hydrocarbon Receptor/Tryptophan Metabolites Signaling |
|  | Aryl Hydrocarbon Receptor Genomic and non-Genomic Signaling |
|  | Model of PPARG signaling in psoriasis |
|  | Model of PPARG related pathways in psoriasis (short version) |

---

|  |  |
| --- | --- |
| MedScan ID | 1018020 |

---

|  |  |
| --- | --- |
| Alias | 5,11-dihydroindolo[3,2-b]carbazole-6-carbaldehyde |
|  | 172922-91-7 |
|  | 229020-82-0 |
|  | 6-formylindolo[3,2-b]carbazole |
|  | FICZ-carbazole |
|  | FICZ |

---

|  |  |
| --- | --- |
| CAS ID | 172922-91-7 |

---

|  |  |
| --- | --- |
| Reaxys ID | 20931887 |

---

|  |  |
| --- | --- |
| InChIKey | OTWFDHQTFRXSAK-UHFFFAOYSA-N |

---

|  |  |
| --- | --- |
| Molecular Formula | C19H10N2O |

---
